# Increased risk of severe neonatal opioid withdrawal syndrome in pregnancies with low placental *ABCB1* DNA methylation

**DOI:** 10.1038/s41372-024-02060-9

**Published:** 2024-07-20

**Authors:** Courtney Townsel, Burnley Truax, Margaret Quaid, Jonathan Covault, Dana C. Dolinoy, Jaclyn M. Goodrich

**Affiliations:** 1 Department of Obstetrics, Gynecology and Reproductive Sciences, University of Maryland, Baltimore, MD, USA.; 2 Department of Environmental Health Sciences, University of Michigan, Ann Arbor, MI, USA.; 3 Department of Psychiatry, University of Connecticut, Farmington, CT, USA.

## Abstract

**BACKGROUND:**

Neonatal opioid withdrawal syndrome (NOWS) is unpredictable. We assessed relationships between placental DNA methylation with in-utero opioid exposure and NOWS severity.

**METHODS:**

Secondary analysis of a prospective multicenter cohort study of pregnancies on methadone or buprenorphine, ≤34 weeks, singleton, 18 or greater. Placental biopsies were collected. Placental DNA methylation levels of *ABCG1, ABCG2, CYP19A1*, and *HSD11B2* were quantified via pyrosequencing following bisulfite conversion. CYP19A1 mRNA levels and umbilical cord drug levels were determined by RT-qPCR and LC-MS respectively. Severe NOWS was diagnosed through Finnegan scoring. *P* value < 0.05 was significant.

**RESULTS:**

Thirty-eight dyads were included. Promoter region methylation for placental *ABCB1* was lower in severe NOWS compared to non-severe NOWS (*p* = 0.04). Placental *CYP19A1* methylation was inversely related to *CYP19A1* mRNA levels and associated with umbilical cord norbuprenorphine levels (*p* < 0.01), but not umbilical cord methadone levels.

**DISCUSSION:**

Lower placental *ABCB1* methylation was associated with severe NOWS. Higher placental *CYP19A1* methylation correlated with higher umbilical cord norbuprenorphine levels.

## INTRODUCTION

Opioid use disorder (OUD) in pregnancy and subsequent neonatal opioid withdrawal syndrome (NOWS) have increased 131% and 82%, respectively, between 2010 and 2017, and both conditions continue to be important public health issues [1]. OUD in pregnancy is associated with opioid overdose and maternal deaths, preterm deliveries, and increased neonatal morbidity [2-5]. Medication for OUD (MOUD), such as methadone and buprenorphine, when used in pregnancy have demonstrated benefit in decreasing maternal mortality. The neonatal impact, however, is unpredictable and leads to prolonged hospital admissions to monitor for the development of severe NOWS. Maternal opioid formulation and dose, length of opioid use, and maternal serum opioid concentrations do not correlate with NOWS severity [6-8]. Inter-individual differences in opioid metabolism and transport within the placenta have been under-explored and may underlie differential NOWS risk. Further, the identification of factors driving NOWS severity is a critical knowledge gap hampering clinical advancement in the prevention and treatment of NOWS and long-term sequelae of prenatal opioid exposure.

**Fig. 1:**
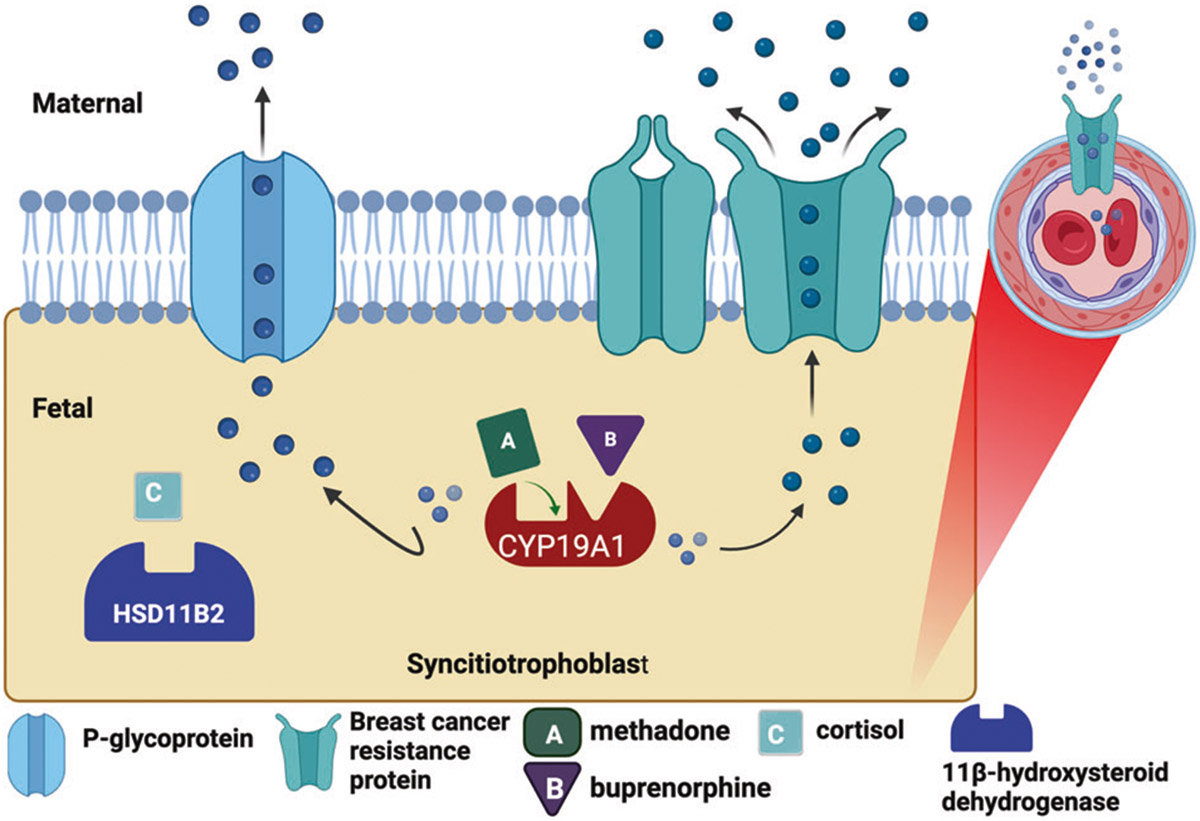
Molecular targets involved in placental regulation of fetal opioid exposure.

The expression and activity of placental drug metabolizing enzymes and placental transporters are key factors in determining fetal exposure to various substances and may play a significant role in controlling fetal opioid exposure. *CYP19A1* encodes aromatase, the major enzyme responsible for metabolizing methadone and buprenorphine, in the human placenta [9-11]. *HSD11β2* (11 beta-Hydroxysteroid dehydrogenase type 2) is an enzyme that plays a crucial role in converting active cortisol to inactive cortisone. Maternal cortisol levels have previously been associated with the development and severity of NOWS [12]. Placental efflux transporters *ABCB1*, which encodes P-glycoprotein (P-gp), and *ABCG2*, which encodes breast cancer resistance protein (BCRP), play an important role in the placental efflux of methadone and buprenorphine back into the maternal circulation, thereby protecting the fetus from exposure to these opioids [13, 14]. Variation in the regulation and function of metabolizing enzymes and these transporters may influence fetal opioid exposure *in utero* and subsequent response in the neonatal period. (Fig. 1).

Our research has previously shown that lower placental *CYP19A1* mRNA expression and lower placental aromatase immunostaining are associated with more severe NOWS [15, 16]. Opioid metabolizing enzymes and efflux transporters are regulated at multiple points, including transcriptional, translational, and post-translational levels. Epigenetics, including DNA methylation, represents an important mechanism in the regulation of gene expression that is relatively stable and heritable across cell divisions. Distinct placental epigenetic profiles have been described for normal and disease-specific obstetric diseases such as preeclampsia [17]. Defining placental epigenetic profiles within MOUD-exposed pregnancies will help us understand inter-individual differences in the transport of opioids, physiological response to opioid exposure, and ultimately its impact on newborn health. The primary aim of this study was to determine the association between DNA methylation of placental candidate genes and NOWS severity. We hypothesized that higher methylation in target genes important for placental transport, opioid metabolism, and stress would be associated with an increased risk of severe NOWS. Our secondary aim was to assess the association between DNA methylation in target genes with fetal umbilical cord drug levels hypothesizing that higher DNA methylation in our target genes would be associated with higher umbilical cord drug levels.

## METHODS

### Design and population

This is a secondary analysis of biospecimen collected during a multi-site prospective cohort study of birthing people prescribed methadone or buprenorphine who delivered at maternity hospitals in the northeast United States between July 2016 and December 2017. Birthing people were included in this analysis if they were 18 years or older, 34 weeks or greater at delivery as determined by last menstrual period and/or earliest ultrasound dating, had a singleton fetus, and were maintained on either methadone or buprenorphine-containing products throughout pregnancy documented in the medical record and confirmed to be on these medications before conception. Patients were excluded if they had missing samples or if neonates had major structural anomalies at birth. Informed consent was obtained from all included patients for the parent study during pregnancy [15]. Maternal and neonatal characteristics were abstracted from the electronic medical record. This study was approved by the University of Michigan institutional review board (HUM00154544/HUM00193721) and all methods were carried out in accordance with STROBE guidelines.

### Placental and umbilical cord samples

Placental samples were collected within 90 min of delivery. Four 1 cm placental tissue samples were collected from just under the fetal side of the placenta, targeting the syncytiotrophoblasts, using either a scalpel or scissors. The samples were washed with normal saline, placed into 2 mL cryotubes containing 1 mL of RNA Later medium, and stored at 4 °C for at least 24 h. Samples were transferred to a freezer at −80 °C until final processing [15].

The details of umbilical cord collection have been described by our team previously [15]. In brief, 6 cm umbilical cord segments were collected from the portion closest to the placental insertion site within 90 min of delivery, washed with normal saline, and stored at −80 °C until sent for drug level analysis. Methadone, buprenorphine, and their metabolite products 2-ethylidene-1,5 dimethyl-3,3 diphenylpyrrolidine (EDDP) and norbuprenorphine were quantified using liquid chromatography-mass spectrometry (LC-MS) at the reference lab United States Drug Testing Lab in Des Plaines, Illinois.

### NOWS assessment

All neonates were assessed using the modified Finnegan scoring system or the Eat-Sleep-Console (ESC) scoring system. In the ESC scoring system infants are scored on their ability to eat 1 oz within an hour, sleep uninterrupted for at least an hour, and be consoled within 10 min. Pharmacotherapy decisions were made according to the delivering institution’s NOWS protocol. Nurses at each institution underwent training twice a year on Finnegan scoring during the study period and ESC training occurred once during the study period when it was rolled out in January 2017. Severe NOWS was diagnosed (1) if the sum of three consecutive Finnegan scores was ≤24 within 72 h of birth [18], (2) two occurrences where at least one ESC criterion is not met, or one instance where all three criteria are not met [19] or (3) if a neonate is treated with a pharmacologic agent for NOWS.

### DNA methylation analysis

Placental samples were partially thawed and tissue was cut using a scalpel and weighed (~14 mg). Duplicates or triplicates were made for each individual participant’s placental sample based on the placental specimen available. DNA was isolated from cut placental tissue using the QIAGEN AllPrep DNA/RNA Micro Kit. DNA concentration was measured via spectrophotometry using a nanodrop. DNA was bisulfite converted using the EpiTect 96 Bisulfite Kit (Qiagen). Primers were designed to amplify unique regions within gene promoters for *ABCB1, CYP19A1, HSD11B2, ABCG2* in bisulfite converted DNA (see Supplementary Table S1). These regions were amplified via PCR, and amplicons were confirmed using a QIAxcel. Next, the amplicons were sequenced via pyrosequencing via a Qiagen PyroMark ID Pyrosequencer which quantifies DNA methylation at site-specific resolution [20, 21]. Briefly, one to three samples per patient were randomized within pyrosequencing plates, and lab technicians were blind to demographics and NOWS severity group. Samples were run in one batch per gene and each batch included negative controls and positive controls of known methylation status. Pyro Q-CpG Software was used to compute percent methylation and perform internal quality control checks to ensure each sample was completely bisulfite converted before analysis, the sample did not deviate from the expected sequence, and the sample generated an adequate amount of signal over background noise. Samples that failed quality control pyrosequencing protocols were excluded from the analysis. Passing replicates were averaged.

### mRNA analysis

To validate methylation findings, we planned to correlate previously derived *CYP19A1* mRNA levels from this cohort to *CYP19A1* methylation levels in this analysis. The mRNA analysis was previously described [15]. In brief, RNA was extracted from placental biopsies and quantified using the NanoDrop. RNA was then reverse-transcribed into cDNA and underwent Real-Time QPCR. Cycle threshold (C_t_) values were obtained and median C_ts_ were used to calculate the Delta-Delta C_t_, the difference between values of reference genes and study samples.

### Statistical analysis

Descriptive statistics were calculated for all participants and stratified by NOWS status. Participant characteristics were compared between the severe NOWS and non-severe NOWS groups via two-sample *t* tests for continuous variables, and chi-square tests or Fischer’s exact test for categorical variables as appropriate. In statistical tests, we used both site-specific DNA methylation and averaged methylation of all CpG sites covered within a given gene. DNA methylation percentage was compared between NOWS severity groups using 2-sample *t* tests. The associations between gene-specific methylation and NOWS severity were measured using unadjusted and adjusted logistic regression models. Maternal variables with differences between NOWS groups (*p* ≤ 0.10) were controlled for in adjusted statistical models. Analyses were also conducted on the full cohort, participants who took buprenorphine and methadone, and a subset of participants in this cohort who took only methadone. Linear regression models were used to model the association of DNA methylation with umbilical cord drug levels, with and without adjustment for age. The Spearman correlation between *CYP19A1* methylation with *CYP19A1* mRNA expression in the placenta obtained previously was also evaluated. Statistical analysis was performed in R Studio Version 2022.12.0 + 353 and SPSS version 29. *P* values < 0.05 were considered statistically significant.

## RESULTS

Thirty-three maternal-infant dyads with MOUD exposure and available biospecimen were included in this analysis. Most of the cohort was non-Hispanic white, identified as smokers, were prescribed methadone, and had Medicaid insurance (Table 1). Infants with severe NOWS had higher peak Finnegan scores, were more likely to be admitted to the neonatal intensive care unit (NICU), and had longer lengths of stay (*p* < 0.01) (Table 1).

### Candidate gene methylation

**Fig. 2:**
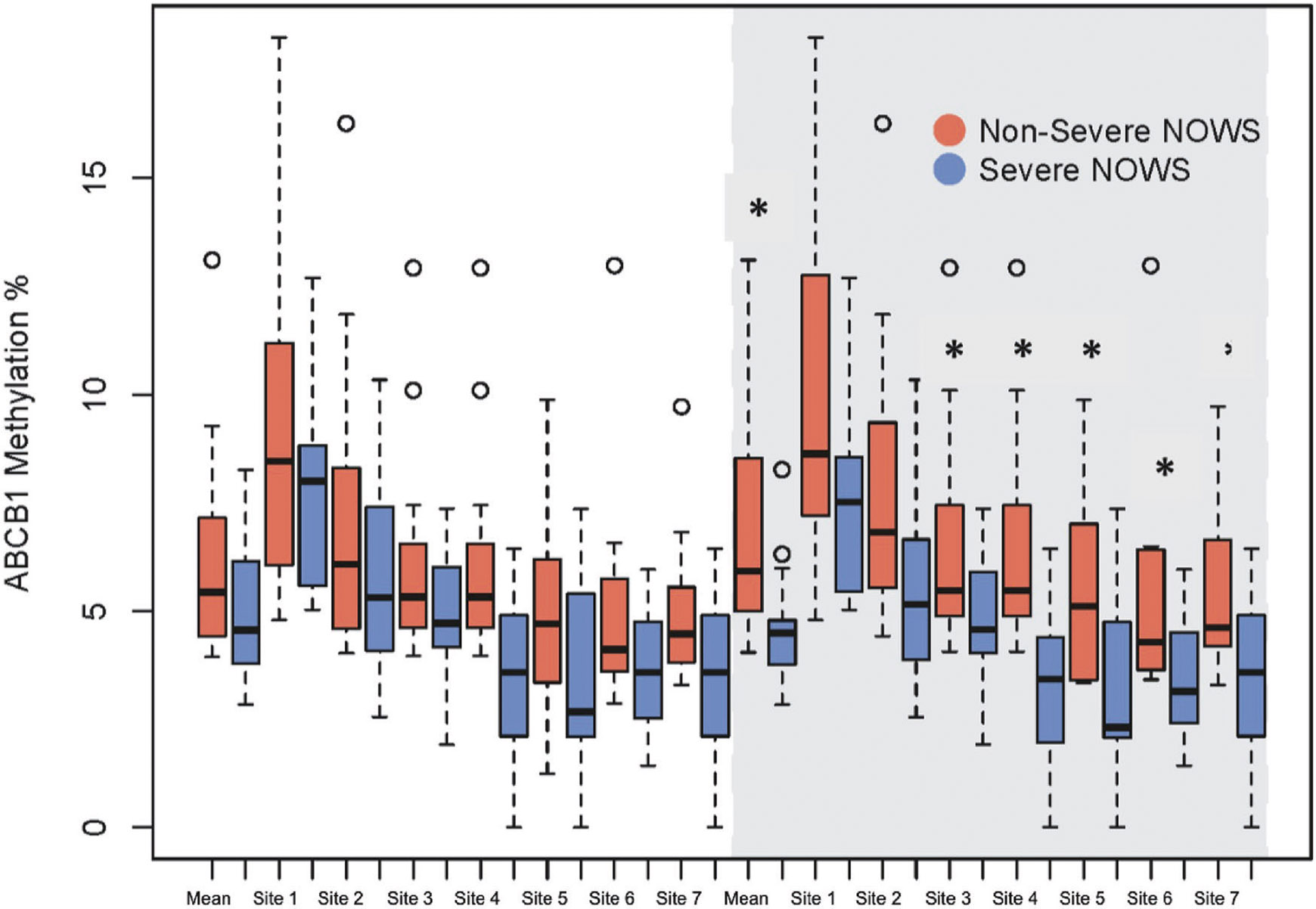
Placental DNA methylation of the *ABCB1* promoter region by NOWS severity in the entire cohort and methadone-exposed pregnancies (shaded in gray). *Significance: *p* value < 0.05.

Overall, placental DNA methylation across candidate genes was low, ranging between 1.5 and 16.6% (Table 2). In bivariate analysis, there were no statistically significant differences in average promoter region DNA methylation between the non-severe and severe NOWS groups for the four candidate genes. (Table 2) When analyzing individual CpG site methylation among the entire cohort, CpG site 7 methylation within the promoter of *ABCB1* was statistically significantly lower in the severe NOWS group compared to the non-severe group (non-severe NOWS 4.9% vs severe NOWS 3.6%, *p* = 0.04) (Table 2). When including only methadone-exposed pregnancies we saw significantly lower methylation in the promoter region of *ABCB1* for the severe NOWS group (non-severe NOWS 6.8% vs severe NOWS 4.6%, *p* = 0.02). When analyzing individual sites within the *ABCB1* promoter in the methadone-exposed cohort, CpG sites 3 through 7 had significantly lower methylation in the severe NOWS group compared to the non-severe NOWS group (*p* < 0.05) (Fig. 2, Table 2). There were no statistically significant differences in CpG site-specific DNA methylation levels between NOWS groups in the methadone-exposed cohort for placental *CYP19A1, ABCG2*, and *HSD11B2* (Table 2).

Unadjusted logistic regression models for *ABCB1* in the methadone cohort showed significantly lower odds of severe NOWS with increased methylation at CpG sites 5 and 7 (site 5: OR 0.57 [95% CI 0.34–0.95] *p* = 0.03; site 7: OR 0.45 [95% CI 0.21–0.96] *p* = 0.04). After adjusting for maternal age at delivery only *ABCB1* CpG site 5 remained statistically significant (site 5: aOR 0.58 [95% CI 0.34–0.99] *p* = 0.04) (Table 3), though effect estimates were similar to the unadjusted model. No relationships between DNA methylation and NOWS were statistically significant in the full cohort in adjusted models.

### Umbilical cord drug analysis

The average maternal methadone dose did not differ between the non-severe NOWS and severe NOWS groups (non-severe NOWS: 107.9 mg ± 38.3 vs severe NOWS: 127.8 mg ± 52.4, *p* = 0.27). The average maternal buprenorphine dose also did not differ between NOWS groups (non-severe NOWS: 11.7 mg ± 4.8 mg vs severe NOWS 16 mg ± 8 mg, *p* = 0.33) (Supplementary Table 2).

There were no significant associations between *ABCB1, ABCG2, CYP19A1*, or *HSD11B2* DNA methylation and umbilical cord methadone or EDDP levels. (Supplementary Table 2) Among the group exposed to buprenorphine, *CYP19A1* methylation was associated with umbilical cord norbuprenorphine levels (OR 0.87 [0.40–1.34], *p* < 0.01). This finding remained statistically significant in a linear regression model after controlling for maternal age (aOR 0.82 [95% CI 0.59–1.06], *p* < 0.01) (Supplementary Table 3). Of note, the sample size for this group is very small (n = 8), and results should be interpreted with caution. There were no other significant associations between candidate gene methylation levels for *ABCB1, ABCG2*, or *HSD11B2* and umbilical cord buprenorphine or norbuprenorphine levels (Supplementary Table 3).

### CYP19A1 methylation and mRNA

*CYP19A1* mRNA data from this cohort was previously analyzed and published [15]. Briefly, *CYP19A1* placental mRNA was noted to be significantly lower in cases of severe NOWS compared to non-severe NOWS. We assessed whether *CYP19A1* DNA methylation levels in the promoter region correlated with *CYP19A1* mRNA levels. *CYP19A1* promoter region methylation was negatively and inversely related to *CYP19A1* mRNA levels, however this finding was not statistically significant (r = −0.13, *p* = 0.48) (Supplementary Table 4).

## DISCUSSION

In this pilot study, we set out to assess whether placental DNA methylation at promoters of key placental transporter, metabolism and stress-responsive genes influence the severity of NOWS and fetal opioid exposure as determined by umbilical cord tissue drug levels. Within our entire cohort, placental *ABCB1* CpG site 7 methylation was significantly lower in the severe NOWS group. When analyzing only the methadone-exposed group, we found lower total placental *ABCB1* methylation and lower CpG site-specific methylation at *ABCB1* sites 3 through 7 in the severe NOWS group. However, after adjustments for potential confounders only the association between placental *ABCB1* site 5 and NOWS severity remained significant. There was a 42% reduction in methylation at *ABCB1* site 5 for cases of severe NOWS in our methadone cohort. When analyzing umbilical cord drug levels, among the buprenorphine group *CYP19A1* methylation was significantly correlated with umbilical cord norbuprenorphine levels. Lastly, there was a weak inverse relationship between placental *CYP19A1* methylation and *CYP19A1* mRNA expression, suggesting functional consequences of epigenetic regulation in this gene.

Methadone is a known substrate of the p-glycoprotein efflux transporter, encoded by *ABCB1*. Variation in this gene may impact fetal opioid exposure and resultant NOWS severity. Hypomethylation of placental *ABCB1* may be associated with reduced expression or activity of this efflux transporter resulting in more fetal opioid exposure and an increased risk of severe NOWS. McLaughlin et al. found higher *ABCB1* methylation in buccal swabs of methadone-exposed neonates compared to unexposed neonates, but found no difference in buccal *ABCB1* methylation between neonates treated and not treated for NOWS (treated for NOWS 18.9% ± 2.8 vs no treatment for NOW 16.6 ± 6.4, *p* = 0.03) [22]. This study was limited by a smaller cohort (*n* = 21) of methadone-exposed pregnancies and may not have been powered to show a difference between NOWS severity groups.

While we did not conduct gene expression analysis of the candidate genes during this study, we previously analyzed *CYP19A1* mRNA in this cohort. Our previous study revealed lower placental *CYP19A1* mRNA in cases of severe NOWS [15]. This analysis shows an inverse relationship between *CYP19A1* methylation and *CYP19A1* mRNA expression, though it was not statistically significant. *CYP19A1* methylation alone, however, did not correlate with NOWS severity. The relationship between *CYP19A1* methylation and mRNA expression supports our overall hypothesis that hypermethylation of *CYP19A1* leads to reduced aromatase mRNA and potentially subsequently reduces aromatase levels and total activity in the placenta resulting in more parent drug in the fetal compartment and a higher risk of severe NOWS. A larger prospective study is ongoing to further analyze this hypothesis.

Previous studies have investigated associations of DNA methylation in other genes with NOWS and NOWS severity. Wachman et al. did not find an association between placental opioid recepter mu 1 (*OPRM1*) methylation and NOWS severity in a cohort of 64 opioid-exposed pregnancies [23]. Earlier work by these authors analyzing buccal and cord blood samples in neonates from opioid-exposed pregnancies revealed a significant increase in *OPRM1* methylation in cases of more severe NOWS (*n* = 86) [24]. These authors analyzed a separate cohort of 58 opioid-exposed pregnancies and found an association between higher neonatal buccal *OPRM1* methylation in infants requiring treatment for NOWS [25]. Radhakrishna et al included 64 opioid-exposed placentas and 32 unexposed placentas in an epigenome-wide association study. The found 1778 significantly differentially methylated CpGs in 1789 genes (FDR < 0.05) [26]. The largest methylation difference was for *BAG2* (+10.5%) for severe NOWS vs non-severe NOWS. Additionally, pathways that were overexpressed in severe NOWS cases included phospholipase C signaling, T cell receptor signaling, Rho family GTPases signaling, cardiac hypertrophy signaling and P53 signlaing pathways [26]. A separate EWAS of opioid exposed (*n* = 19) and unexposed (*n* = 20) pregnancies found 258 differentially methylated genes in placenta [27]. The enrichment analysis identified epigentic dysregulation of genes involved in synaptic structure, chemical synaptic transmission and nervous system development [27]. This cohort was too small to assess epigenetic differences within opioid-exposed group. Additionally, both of these epigenome-wide association studies included a variety of opioid medication exposures including methadone, buprenorphine, fentanyl and “other prescribed opioids” limiting generalizability.

While there has been growing interest in the genetics and epigenetics of opioid exposure and response in pregnancy there has been great heterogeneity in the tissue of interest studied. Few studies have focused on the placenta. Our work has centered on epigenetic regulation within the placenta as a sentinel factor influencing fetal opioid exposure and NOWS severity. The placenta is the most critical organ in pregnancy with a major function of protecting the developing fetus from deleterious exposures. We urge other investigators to strongly consider incorporating the placenta into models of maternal-fetal exchange to fully characterize relationships between exposures, pregnancy, and birth outcomes.

There are many strengths to this study. We conducted a hypothesis-driven approach to investigate placental DNA methylation at key genes important for placental metabolism, transport and stress response and their association with clinical outcomes and fetal opioid exposure. The study was conducted in a MOUD-exposed pregnant population with strong clinical data. We are continuing to add knowledge about the molecular underpinnings of opioid-exposed pregnancies. We have identified potential markers of NOWS severity in pregnancies near delivery. There are a few weaknesses of this study as well. The first is our small sample size, especially when conducting analyses stratified by opioid type. We were also limited to mRNA expression data of only one of the analyzed genes. We recognize that both Finnegan scoring and the newly introduced Eat, Sleep, Console scoring used to define the primary outcome are limited by inter-operator variability. These were the standards of care, however during the study period. Additionally, although we had short-term neonatal outcome data we did not have long-term infant outcomes to link our findings with clinically meaningful milestones beyond delivery hospital discharge. Finally, our hypothesis-driven approach to selecting promoter regions of only four candidate genes is both a strength and limitation since other genes could be contributing to NOWS severity. In addition to other genes and gene regions, epigenetic modifications beyond DNA methylation also influence placental gene expression and consequently fetal opioid exposure and response.

In summary, we provide new insights into the placental methylome in pregnancies exposed to methadone and buprenorphine among 4 candidate genes. Unique placental methylation profiles near delivery may be indicative of fetal opioid exposure and NOWS severity risk. Contrary to our hypothesis, reduced gene methylation of *ABCB1* was associated with more severe NOWS. This may suggest normal protein product formation of p-glycoprotein, but altered function reducing efflux of opioids from the fetal compartment back to the maternal compartment leading to a more severe NOWS outcome. Future studies should seek to analyze placental methylation, mRNA and protein expression, and enzyme or transporter activity in conjunction with short and long-term clinical outcomes. Additionally, placental methylome variation for these genes should be defined in unexposed pregnancies to assess the impact of methadone and buprenorphine exposure alone on the placental methylome. We have an ongoing prospective case-control study to address these gaps. Future works will seek to assess the entire placental methylome for other novel candidate genes that may impact fetal opioid exposure and NOWS severity with the hope of linking this prenatal exposure to long-term neurodevelopmental outcomes.

## Supplementary Material

Supplementary Table 1

Supplementary Table 2

Supplementary Table 4

Supplementary Table 3

STROBE Statement

## Figures and Tables

**Table 1.** Maternal and neonatal demographics in MOUD-exposed cohort by neonatal opioid withdrawal syndrome (NOWS) severity.

	Total cohort (*n* = 33)	Non-severe NOWS (*n* = 16)	Severe NOWS (*n* = 17)	*p* value
Maternal characteristics				
Maternal age (years)	30.1 ± 3.9	31.8 ± 4.0	28.5 ± 3.2	<0.01*
Gravida	2.9 ± 1.6	3.0 ± 1.4	2.9 ± 1.8	0.83
Race				
White	31 (93.9%)	16 (100%)	15 (88.2%)	0.86
African American	1 (3.0%)	0	1 (5.9%)	NA
Other	1 (3.0%)	0	1 (5.9%)	NA
Ethnicity				
Non-Hispanic	29 (87.9%)	15 (93.8%)	14 (82.4%)	0.85
Hispanic	4 (12.1%)	1 (6.3%)	3 (17.7%)	NA
MOUD				
Methadone	25(75.8%)	10 (62.5%)	15 (88.2%)	0.32
Buprenorphine	8 (24.4%)	6 (37.5%)	2 (11.8%)	NA
Smoker	24 (72.7%)	10 (62.5%)	14 (82.4%)	0.41
Length of stay (days)	4.0 ± 1.1	4.1 ± 1.0	3.8 ± 1.2	0.45
Cesarean	15 (45.5%)	8 (50.0%)	7 (41.2%)	0.80
Medicaid Insurance	31 (93.9%)	14 (87.5%)	17 (100%)	0.59
Neonatal characteristics				
Gestational age at delivery (weeks)	38.5 ± 1.6	38.6 ± 1.8	38.5 ± 1.4	0.95
Gestational weight (grams)	3051 ± 549	3069 ± 512	3034 ± 596	0.86
Male	18 (54.6%)	9 (56.3%)	9 (56.3%)	1
NICU admission for NOWS	15 (45.5%)	2 (12.5%)	13 (76.5%)	<0.01*
Peak Finnegan Score	10.2 ± 4.0	7.2 ± 1.9	13 ± 3.3	<0.01*
Length of stay (days)	10.9 ± 6.7	7.9 ± 3.1	14.0 ± 7.9	<0.01*

* Significance level <0.05.

*T* test on continuous variables, chi-squared test on categorical variables.

*NOWS* neonatal opioid withdrawal syndrome, *MOUD* medication for opioid use disorder, *NICU* neonatal intensive care unit.

**Table 2.** Mean gene and CpG site DNA methylation by NOWS severity among the overall cohort and the methadone cohort.

	Overall cohort (*n* = 33)			Methadone cohort (*n* = 25)		
	Non-severe NOWS	Severe NOWS	*P* value	Non-severe NOWS	Severe NOWS	*P* value
ABCB1	6.20 (2.5)	4.94 (1.6)	0.10	6.77 (2.78)	4.64 (1.41)	0.02*
Site 1	9.09 (3.79)	7.92(2.4)	0.31	9.77 (4.18)	7.41 (2.1)	0.13
Site 2	7.11 (3.33)	5.81 (2.19)	0.20	7.87 (3.71)	5.42 (2.05)	0.05
Site 3	6.11(2.38)	4.88 (1.56)	0.09	6.69 (2.79)	4.74 (1.6)	0.04*
Site 4	6.45 (2.05)	5.32 (1.68)	0.10	6.89 (2.39)	5.06 (1.6)	0.03*
Site 5	4.75 (2.22)	3.37 (2.07)	0.08	5.4 (2.12)	3.07 (2.04)	0.01*
Site 6	4.95 (2.45)	3.67 (1.38)	0.08	5.38 (2.9)	3.47 (1.34)	0.04*
Site 7	4.93 (1.66)	3.62 (1.73)	0.04*	5.36 (1.9)	3.34 (1.66)	0.01*
CYP19A1	16.6 (3.46)	17.85 (4.51)	0.37	17.48 (3.75)	18.24 (4.67)	0.66
Site 1	14.91 (3.42)	16.74 (4.74)	0.21	15.78 (3.75)	17.15 (4.89)	0.44
Site 2	16.90 (3.92)	18.07 (4.76)	0.45	17.84 (4.39)	18.46 (4.92)	0.75
Site 3	15.50 (3.17)	16.31 (4.18)	0.54	16.34 (3.53)	16.68 (4.32)	0.83
Site 4	19.83 (4.12)	20.83 (4.77)	0.52	20.8 (4.33)	21.16 (5)	0.85
Site 5	15.82 (3.09)	17.31 (4.57)	0.28	16.65 (3.2)	17.74 (4.7)	0.50
HSD11B2	3.30 (0.87)	3.36 (1.32)	0.89	3.69 (0.71)	3.21 (1.30)	0.24
Site 1	4.91 (0.94)	4.64 (1.61)	0.56	5.37 (0.77)	4.51 (1.68)	0.10
Site 2	0.62 (0.71)	0.87 (1.08)	0.44	0.69 (0.78)	0.68 (0.88)	0.97
Site 3	5.06 (1.16)	5.31 (1.73)	0.63	5.58 (0.76)	5.13 (1.72)	0.38
Site 4	2.23 (1.21)	1.91 (1.59)	0.52	2.68 (1.21)	1.79 (1.66)	0.13
Site 5	3.70 (1.44)	4.07 (1.62)	0.50	4.14 (1.39)	3.91 (1.65)	0.72
ABCG2	1.49 (1.05)	2.04 (1.57)	0.24	1.67 (2.15)	1.23 (1.65)	0.41
Site 1	2.5 (1.22)	3.97 (4.37)	0.20	2.55 (1.5)	4.11 (4.65)	0.24
Site 2	1.48 (0.97)	1.82 (1.24)	0.38	1.71 (1.14)	1.91 (1.3)	0.69
Site 3	0.69 (0.87)	1.16 (1.27)	0.22	0.72 (1)	1.23 (1.34)	0.28
Site 4	1.76 (1.92)	1.42 (1.62)	0.75	1.82 (1.52)	2 (1.72)	0.80
Site 5	0.75 (1.04)	1.07 (1.18)	0.42	0.99 (1.21)	1.16 (1.22)	0.74
Site 6	1.76 (1.76)	2.32 (1.65)	0.31	2.2 (1.63)	2.5 (1.68)	0.67

% (SD).

Significance: *p* value < 0.05.

* Significance level <0.05.

Two-sided *t* test.

**Table 3.** Unadjusted and adjusted models for *ABCB1* methylation and severe NOWS in the overall cohort and methadone cohort.

	Overall cohort			Methadone cohort		
	Unadjusted odds ratio severe NOWS	95% CI	*P* value	Unadjusted odds ratio severe NOWS	95% CI	*P* value
*ABCB1*	0.70	0.45–1.09	0.11	0.54	0.29–1.02	0.06
*site 1*	0.88	0.7–1.12	0.30	0.77	0.56–1.06	0.11
*Site 2*	0.83	0.62–1.11	0.21	0.70	0.47–1.05	0.09
*Site 3*	0.69	0.43–1.10	0.12	0.60	0.34–1.07	0.08
*Site 4*	0.70	0.44–1.09	0.12	0.58	0.32–1.04	0.07
*Site 5*	0.73	0.51–1.05	0.09	0.57	0.34–0.95	0.03*
*Site 6*	0.63	0.36–1.10	0.10	0.51	0.24–1.07	0.07
*Site 7*	0.59	0.34–1.01	0.06	0.45	0.21–0.96	0.04*
	Adjusted odds ratio severe NOWS	95% CI	*P* value	Adjusted odds ratio severe NOWS	95% CI	*P* value
*ABCB1*	0.73	0.46–1.17	0.19	0.56	0.29–1.08	0.09
*site 1*	0.93	0.72–1.20	0.58	0.8	0.57–1.12	0.20
*Site 2*	0.85	0.62–1.16	0.30	0.72	0.48–1.10	0.13
*Site 3*	0.64	0.35–1.17	0.15	0.61	0.33–1.12	0.11
*Site 4*	0.74	0.46–1.17	0.19	0.62	0.34–1.12	0.11
*Site 5*	0.73	0.49–1.09	0.12	0.58	0.34–0.99	0.04*
*Site 6*	0.63	0.34–1.19	0.16	0.53	0.25–1.13	0.10
*Site 7*	0.61	0.33–1.11	0.10	0.47	0.21–1.02	0.06

Model adjusted for maternal age.

* Significance *p* < 0.05.

## Data Availability

Data will be made available upon reasonable request.
