# Supplementary material for: Increased risk of severe neonatal opioid withdrawal syndrome in pregnancies with low placental ABCB1 DNA methylation
Source: J Perinatol. 2024 Jul 20;45(4):458–64. doi: 10.1038/s41372-024-02060-9 (PMC11743817; doi:10.1038/s41372-024-02060-9)
Supplement: Supplementary file 1 — Supplementary Table 1 [file 41372_2024_2060_MOESM1_ESM.docx]

Supplement Table 1: Primer sequences and pyrosequencing assay details

| **Gene Name** | **Forward primer** | **Reverse Primer** | **Sequencing Primer** | **Sequence to Analyze in PyroMark** | **Amplicon length (bp)** | **# CpG sites** | **Amplicon location** | **PCR Conditions** |  |  |  |  |
| --- | --- | --- | --- | --- | --- | --- | --- | --- | --- | --- | --- | --- |
| *ABCB1* | TAAGTGGGGTTA  GATTTAGATTTAGG | biotin-CCATTACCAT  CCCCTTCAAAATCCAT | TTAAATAGTAGTGGTATTGGATTAT | GTTGTTYGGA GYGYGTATAG TTYGYGYGGT  GYGGGGATTT GTTTTTTGAG TT | 456 | 7 | chr7: 87600159-87600614 | 15 minutes at 95℃ then [45 cycles X (95℃ for 30 seconds, 58℃ for 30 seconds, 72℃ for 60 seconds)] then 72℃ for 10 minutes | | | | |
| *ABCG2* | AGGGAGGGAG  GTGGTATTAG | biotin-AACCCCTTCC  CCCAACCA | AGGGAGGTGGTATTAGT | T TTGTTGGYG GTTTAGYGYG GTAGGATAYG  TGTGYGTTTT TAGTYGGGT | 373 | 5 | chr4: 88158705-88159077 | 15 minutes at 95℃ then [45 cycles X (95℃ for 30 seconds, 56℃ for 30 seconds, 72℃ for 60 seconds)] then 72℃ for 10 minutes | | | | |
| *CYP19A1* | TGGAGGTAAATA  GGAAGGTGAAGA | biotin-ACCTAAAAAAA  ATATCTAATCCCACAAAT | AGGAAGGT  GAAGAAGA | ATTTATTTTA TTAGGAYGGA AGGTTTTGTG TTYGGGATTT TTTAGAYGTY  GYGTATGTAT TTTTTAATTT GATTGAG | 139 | 5 | chr 15: 51338435-51338573 | 15 minutes at 95℃ then [45 cycles X (95℃ for 30 seconds, 58℃ for 30 seconds, 72℃ for 60 seconds)] then 72℃ for 10 minutes | | | | |
| *HSD11B2* | TTAAGTTTTGGAA  GGAAAGGGAAAGA | Biotin-ACATCCCCAT  ACCCTTTACTAATC | AGTTTTTGTTTTAGGTAGG | TTTTGTGGTY GYGAGTTGTT TAGGGGTGAG  YGYGTTTTAG GGTGTGTGYG TTAGGGATTT | 307 | 5 | chr16: 67430512-67430745 | 15 minutes at 95℃ then [45 cycles X (95℃ for 30 seconds, 54℃ for 30 seconds, 72℃ for 60 seconds)] then 72℃ for 5 minutes |  |  |  |  |
