# Supplementary material for: Increased risk of severe neonatal opioid withdrawal syndrome in pregnancies with low placental ABCB1 DNA methylation
Source: J Perinatol. 2024 Jul 20;45(4):458–64. doi: 10.1038/s41372-024-02060-9 (PMC11743817; doi:10.1038/s41372-024-02060-9)
Supplement: Supplementary file 2 — Supplementary Table 2 [file 41372_2024_2060_MOESM2_ESM.docx]

**Supplemental Table 2. Maternal and Umbilical Cord Levels of Medication for Opioid Use Disorder and Metabolites**

|  | **Non-Severe NOWS (n=13)** | **Severe NOWS (n=15)** | **P-value** |
| --- | --- | --- | --- |
| Methadone Dose (mg) | 107.9 (38.3) | 127.75 (52.4) | 0.25 |
| Umbilical Cord EDDP (ng/g) | 51.1 (31.8) | 110.2 (109.6) | 0.06 |
| Umbilical Cord Methadone (ng/g) | 138.6 (51.1) | 157.9 (78.4) | 0.44 |
| Umbilical Cord Methadone:EDDP | 3.9 (2.9) | 2.3 (1.5) | 0.09 |
|  |  |  |  |
|  | **Non-Severe NOWS (n=6)** | **Severe NOWS**  **(n=3)** |  |
| Buprenorphine Dose (mg) | 11.7 (4.8) | 16 (8) | 0.46 |
| Umbilical Cord Norbuprenorphine (ng/g) | 2 (1.7) | 3.1 (0.4) | 0.25 |
| Umbilical Cord Buprenorphine (ng/g) | 1.2 (0.6) | 1.0 (0.7) | 0.66 |
| Umbilical Cord Buprenorphine: Norbuprenorphine | 1.1 (1.0) | 0.4 (0.3) | 0.23 |

Number (SD)

*Significance: p-value < 0.05.

NOWS: neonatal opioid withdrawal syndrome

EDDP: 2-ethylidene-1,5 dimethyl-3,3 diphenylpyrrolidine
