# Supplementary material for: Increased risk of severe neonatal opioid withdrawal syndrome in pregnancies with low placental ABCB1 DNA methylation
Source: J Perinatol. 2024 Jul 20;45(4):458–64. doi: 10.1038/s41372-024-02060-9 (PMC11743817; doi:10.1038/s41372-024-02060-9)
Supplement: Supplementary file 3 — Supplementary Table 3 [file 41372_2024_2060_MOESM3_ESM.docx]

| **Supplemental Table 3. Unadjusted and Adjusted Linear Regression Model for Candidate Gene Methylation and Umbilical Cord Drug Levels** | | | | | | |  |
| --- | --- | --- | --- | --- | --- | --- | --- |
| **Unadjusted Model** | **Umbilical EDDP (ng/g)** | | | **Umbilical Methadone (ng/g)** | | |  |
|  | **Estimate** | **95% CI** | **P-value** | **Estimate** | **95% CI** | **P-value** |  |
| *ABCB1* | -6.93 | -24.4 - 10.5 | 0.42 | -0.78 | -14.2 – 12.6 | 0.91 |  |
| *CYP19A1* | 0.08 | -9.6 – 9.7 | 0.99 | 2.22 | -5.0 – 9.4 | 0.53 |  |
| *HSD11B2* | -0.34 | -38.0 – 37.3 | 0.98 | -2.78 | -31.2 – 25.7 | 0.84 |  |
| *ABCG2* | -1.61 | -29.6 – 26.4 | 0.91 | 8.15 | -12.8 – 29.1 | 0.43 |  |
| **Unadjusted Model** | | **Umbilical Norbuprenophine (ng/g)** | | | **Umbilical Buprenorphine (ng/g)** | | |
|  | | **Estimate** | **95% CI** | **P-value** | **Estimate** | **95% CI** | **P-value** |
| *ABCB1* | | 0.15 | -1.07 – 1.37 | 0.75 | 0.08 | -0.29 – 0.44 | 0.63 |
| *CYP19A1* | | 0.87 | 0.40-1.34 | <0.01* | 0.12 | -0.12 – 0.37 | 0.27 |
| *HSD11B2* | | 0.65 | -1.74 – 3.05 | 0.49 | 0.13 | -0.38 – 0.64 | 0.55 |
| *ABCG2* | | -1.91 | -4.65 – 0.83 | 0.13 | 0.25 | -0.85 – 1.34 | 0.60 |

| **Adjusted Model^α^** | **Umbilical Norbuprenorphine (ng/g)** | | |
| --- | --- | --- | --- |
|  | **Estimate** | **95% CI** | **P-value** |
| *ABCB1* | -0.11 | -1.82 – 1.54 | 0.85 |
| *CYP19A1* | 0.82 | 0.59 – 1.06 | <0.01* |
| *HSD11B2* | 0.21 | -3.50 – 3.92 | 0.87 |
| *ABCG2* | -1.82 | -4.79 – 1.15 | 0.14 |

Estimate from unadjusted and adjusted model.

α:Adjusted model includes maternal age

*Significance: p-value < 0.05.
