# Supplementary material for: Increased risk of severe neonatal opioid withdrawal syndrome in pregnancies with low placental ABCB1 DNA methylation
Source: J Perinatol. 2024 Jul 20;45(4):458–64. doi: 10.1038/s41372-024-02060-9 (PMC11743817; doi:10.1038/s41372-024-02060-9)
Supplement: Supplementary file 4 — Supplementary Table 4 [file 41372_2024_2060_MOESM4_ESM.docx]

**Supplemental Table 4:** Correlation between placental *CYP19A1* methylation and *CYP19A1* mRNA levels

|  | **Spearman’s rho** | **p-value** |
| --- | --- | --- |
| *CYP19A1* | -0.13 | 0.48 |
| CpG Site 1 | -0.16 | 0.39 |
| CpG Site 2 | -0.12 | 0.51 |
| CpG Site 3 | -0.17 | 0.36 |
| CpG Site 4 | -0.13 | 0.47 |
| CpG Site 5 | -0.18 | 0.32 |

*Significance: p-value < 0.05
